# Balloon-Assisted and Snare-Facilitated Retrieval of a Kinked Radial Catheter Entrapped in the Axillary Artery

**DOI:** 10.1016/j.jaccas.2026.108133

**Published:** 2026-06-10

**Authors:** Saliha Erdem, Mangesh Kritya, Hanad Bashir, Chloe Kharsa, Karl Abou Zeid, Masroor A. Khan, Joe Aoun

**Affiliations:** Department of Cardiology, Houston Methodist Hospital, Houston, Texas, USA

**Keywords:** complication, myocardial infarction, percutaneous coronary intervention

## Abstract

**Background:**

Catheter kinking during transradial coronary angiography is rare but may result in fixed entrapment, where forceful manipulation risks vascular injury.

**Case Summary:**

A 67-year-old woman with non–ST-segment elevation myocardial infarction underwent diagnostic coronary angiography via right radial access using a 6-F FL3.5 catheter. During manipulation, the catheter became fixed and kinked in the axillary artery, with loss of rotational control. Standard wire-based maneuvers failed. Dual arterial access was obtained with femoral snare stabilization of the distal catheter tip and intraluminal low-pressure balloon inflation to straighten the kink, allowing telescoping guide catheter advancement and controlled retrieval. Final angiography showed no vascular injury. The thrombotic culprit lesion was treated with aspiration thrombectomy and balloon angioplasty.

**Discussion:**

This case demonstrates stepwise application of a bailout technique to manage complex radial catheter entrapment.

**Take-Home Message:**

Early recognition and controlled escalation using dual access and intraluminal support can enable safe catheter retrieval without treatment delays.

## Visual Summary

**Figure:**
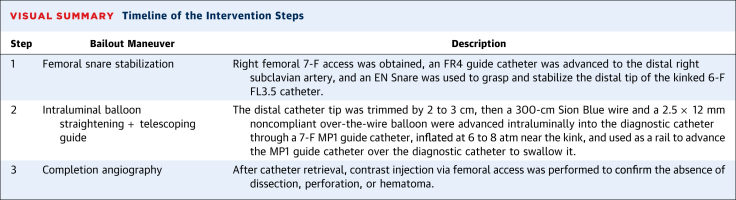

Catheter kinking during transradial coronary angiography is uncommon, but when it occurs in the brachial or axillary artery it can become fixed and nonrotatable, complicating safe extraction.^1^^,^^2^ True kinking requiring combined snare via femoral and balloon-assisted rescue through a transradial approach is rarely reported.^3^ Here we describe a stepwise, minimally invasive technique that successfully restored catheter geometry and allowed safe retrieval without vascular injury. This case adds to the limited literature on managing complex radial access catheter complications.

**Take-Home Message**

• Early recognition and controlled escalation using dual access and intraluminal support can enable safe catheter retrieval without delaying the treatment.

## Patient Information

A 67-year-old woman presented with chest pain and rising high-sensitivity troponin levels, consistent with non–ST-segment elevation myocardial infarction (NSTEMI). Relevant history included nonischemic cardiomyopathy and chronic kidney disease stage 3a. She was hemodynamically stable, with no signs of heart failure or limb ischemia. Serial electrocardiograms showed left bundle branch block and evolving ST-T changes.

## Therapeutic Intervention

Right radial access was obtained with a 6/7-F slender sheath, and a 6-F FL3.5 diagnostic catheter was advanced for coronary angiography. During manipulation in the subclavian-axillary segment, counterclockwise torque resulted in catheter kinking, with loss of pushability and rotational control. Attempts to restore catheter geometry with clockwise rotation and using a Wholey wire, Glidewire, and 0.014-inch coronary wire followed by an Amplatz wire were unsuccessful. Given the risk of vascular injury with forceful traction, a stepwise bailout strategy was undertaken.

### Step 1: Secondary femoral access and snaring

**Figure 1:**
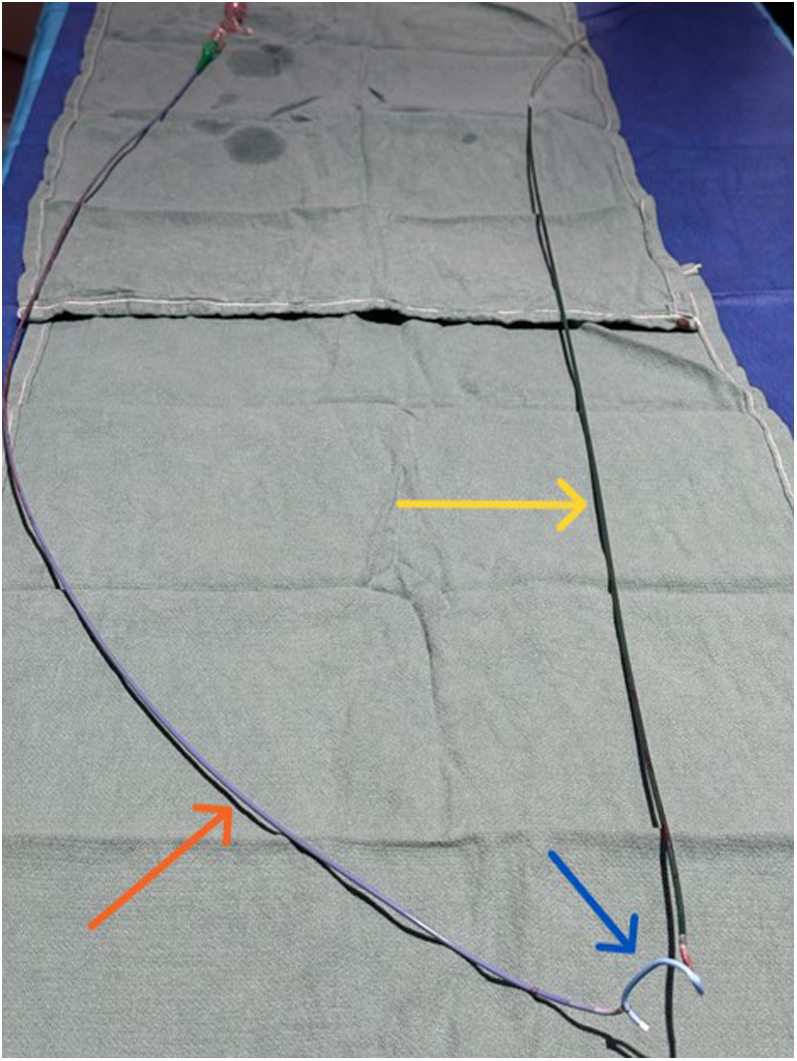
Equipment Used Shown are the EN Snare (orange arrow), 6-F kinked diagnostic catheter (blue arrow), and 7-F MP1 guide catheter (yellow arrow).

A right femoral 7-F sheath was placed under ultrasound guidance (Figure 1). A 6-F FR4 guide catheter was advanced into the distal right subclavian artery. Through it, an EN Snare device (Video 1) (Merit Medical) was positioned to grasp the distal tip of the kinked FL3.5 catheter (Video 2).

### Step 2: Balloon-assisted mother-daughter technique

The 6-F FL3.5 diagnostic catheter tip was trimmed by approximately 2 to 3 cm at the end (Video 3). Outside the body, a long 300-cm Sion Blue wire (Asani Intecc) was advanced through a 2.5 × 12 mm noncompliant balloon (over-the-wire balloon), both of which were advanced into a 7-F MP1 guide catheter. After that, the Sion Blue and over-the-wire balloon were advanced into the diagnostic catheter (Video 4). The balloon was inflated close to the kink, and the inflation pressure was limited to 6-8 atm. The wire and balloon served as a rail to advance the MP1 guide catheter over the diagnostic catheter using the mother-daughter technique. Once the MP1 catheter reached the distal tip of the diagnostic catheter, the EN Snare was released (Video 5). The whole system was taken out except the MP1 guide catheter.

### Step 3: Controlled withdrawal and confirmation of arterial integrity

The MP1 guide catheter (Video 6) was subsequently removed over the Wholey wire (Medtronic). Contrast injection through the 6-F FR4 catheter (via femoral access) demonstrated no evidence of vascular injury, dissection, or hematoma. The FR4 catheter was then positioned into the ascending aorta, and selective coronary angiography was completed without further complications (Video 7).

### Step 4: Treatment of the culprit coronary lesion

After successful catheter retrieval and confirmation of vascular integrity, selective coronary angiography was completed. Angiography demonstrated a large thrombus burden in the distal right coronary artery, consistent with the culprit lesion for the patient's NSTEMI, with baseline TIMI flow grade 0 distally.

The lesion was treated with mechanical aspiration thrombectomy using the Indigo system (Penumbra), supplemented by manual aspiration, intracoronary and intravenous eptifibatide, distal vasodilator therapy, and low-profile balloon angioplasty (2.0 × 12 mm and 2.0 × 20 mm). Intravascular ultrasound confirmed adequate luminal expansion without residual dissection or plaque rupture. Final angiography demonstrated TIMI flow grade 3 in the distal right coronary artery and posterolateral branch, with persistent TIMI flow grade 0 in the distal posterior descending artery despite aggressive thrombectomy.

The catheter entrapment and retrieval sequence was managed efficiently and did not result in a clinically significant delay in identifying or treating the NSTEMI culprit lesion.

## Follow-Up and Outcomes

Hemostasis was achieved via TR Band (Terumo) and manual femoral compression. The patient resumed systemic anticoagulation with intravenous heparin after sheath removal. She was discharged on medical therapy with no vascular sequelae.

## Discussion

Catheter kinking during transradial coronary angiography is uncommon. Most published reports describe catheter knotting or guidewire entrapment as rare complications. It typically occurs when a guide catheter is aggressively maneuvered in a tortuous access vessel.^2^ Knotting is the best-characterized complication and is often managed with gentle traction, stiff-wire straightening, or isolated snare retrieval.^2^ In contrast, axillary catheter kinking occurs when the catheter loses coaxial alignment, becomes fixed against arterial curvature, and cannot be rotated or withdrawn.^1^^,^^3^^,^^4^

Minor catheter kinking is often manageable with basic maneuvers, including light traction, catheter rotation, or guidewire passage.^5^ Yet, when the kink forms a complex loop, these simple methods are frequently ineffective.^5^ In our case, an 0.014-inch wire, a Wholey wire, and an Amplatz wire failed to restore the lumen or reverse the torque effect. Traction using a snare and reversing the torque was also not helpful. Also, uncontrolled traction in the axillary segment might cause significant complication such as risk of arterial dissection, perforation, or avulsion**,** and might require surgical consultation.

Snare-based stabilization from femoral access has been described for knotted or entrapped catheters and can provide counter-traction.^1^, ^2^, ^3^ Balloon-based rescue techniques have also been reported to straighten kinked catheters or trap a device within a guide.^6^^,^^7^ In the present case, we applied these maneuvers in a sequential manner. After snare stabilization of the distal catheter tip, a small noncompliant balloon was advanced *inside* the kinked diagnostic catheter to maintain access to the catheter and (create a rail) over which advancing a larger guide over the “cut” diagnostic catheter to swallow it (mother-daughter technique). Final angiography confirmed no vascular damage**,** underscoring the safety of this stepwise escalation strategy.

A practical escalation strategy is to stop torque immediately, attempt wire-based straightening, obtain secondary access for snare stabilization if needed, consider balloon-assisted straightening with a telescoping guide catheter, and perform completion angiography to exclude vascular injury. This stepwise approach may help avoid surgical retrieval.

## Conclusions

Axillary catheter kinking can occur during transradial angiography and may be difficult to resolve with standard wire-based maneuvers. In selected cases, femoral snare stabilization and balloon-assisted straightening can be used to support controlled catheter retrieval. A structured escalation strategy may help minimize complications.

## Uncited Figure

Figure 1

## Funding Support and Author Disclosures

The authors have reported that they have no relationships relevant to the contents of this paper to disclose.

## Appendix

Video 1 Video Describing the Technique

Video 2 A 6-F FR4 Catheter Was Advanced Into the Distal Right Subclavian Artery

Video 3A An Amplatz GooseNeck Snare (10- to 15-mm loop) was positioned to grasp the distal tip of the kinked FL3.5 catheter.

Video 3B An Amplatz GooseNeck Snare (10- to 15-mm loop) was positioned to grasp the distal tip of the kinked FL3.5 catheter.

Video 4 From the Radial Side, a Sion Blue Wire Was Readvanced Through the Kinked FL3.5 Catheter The catheter tip was trimmed to allow passage of a 7-F MP1 guide catheter.

Video 5 The MP1 Guide Catheter Was Advanced to the Distal Tip of the FL3.5 (Swallowing the Diagnostic Catheter) The balloon was then deflated.

Video 6 Coordinated Traction Was Applied: The Snare Was Gently Withdrawn Into the Femoral Sheath While the Straightened FL3.5 Catheter Was Pulled Back Over the Sion Blue Wire From the Radial Side The catheter was removed without resistance or fragmentation.

Video 7 Contrast Injection Through the 6-F FR4 Catheter (via Femoral Access) Demonstrated No Evidence of Vascular Injury, Dissection, or Hematoma

## References

[1] Kim J.Y., Moon K.W., Yoo K.D. (2012). Entrapment of a kinked catheter in the radial artery during transradial coronary angiography. J Invasive Cardiol.

[2] Ben-Dor I., Rogers T., Satler L.F., Waksman R. (2018). Reduction of catheter kinks and knots via radial approach. Catheter Cardiovasc Interv.

[3] Khoubyari R., Arsanjani R., Habibzadeh M.R., Echeverri J., Movahed M.R. (2012). Successful removal of an entrapped and kinked catheter during right transradial cardiac catheterization by snaring and unwinding the catheter via femoral access. Cardiovasc Revasc Med.

[4] Aminian A., Fraser D.G., Dolatabadi D. (2015). Severe catheter kinking and entrapment during transradial coronary angiography: percutaneous retrieval using a sheathless guide catheter. Catheter Cardiovasc Interv.

[5] Elrayes M.M., Al-Ogaili A., Jalli S., Brilakis E.S. (2024). Prevention and treatment of coronary catheter kinking. Catheter Cardiovasc Interv.

[6] Tüner H., Polat F., Kayhan Ö., Göktekin Ö. (2025). Femoral access-assisted balloon trapping: a novel approach to managing kinked catheter during transradial coronary Intervention-A case report. Catheter Cardiovasc Interv.

[7] Rahimov U., Karimli E., Musayeva G., Salimov Z. (2022). Rescue of severely twice-kinked catheter during transradial coronary intervention using balloon technique. Cardiovasc Revasc Med.

